# Supplementary material for: Planning and Presenting Workshops That Work: A Faculty Development Workshop
Source: MedEdPORTAL. 2021 May 11;17:11158. doi: 10.15766/mep_2374-8265.11158 (PMC8110637; doi:10.15766/mep_2374-8265.11158)
Supplement: Supplementary file 1 — Facilitator Guide.docxSession Agenda.docWorkshop Slides.pptWorkshop Template Handout.docxAdditional Handout.docxAdvanced Handout.docxSession Evaluation.docx [file mep_2374-8265.11158-s001.zip › D. Workshop Template Handout.docx]

**Workshop Template based on Kolb’s Learning Cycle**

**Topic** __________________________________________________________________

**Length** ___________ **Target audience** __________________________________

| Goals |  |
| --- | --- |
| Objectives | 1.  2.  3. |
| **Ice breaker/reflection/**  **attention getter** |  |
| **Share/Didactics** |  |
| **Practice/Activities** |  |
| **Apply what was learned (Get the participants to make a commitment to try this at “home” or to reflect on how they can use this)** |  |
| **Summary/Conclusion** |  |

**AV equipment needed:**

**Handouts:**

**WORKSHOP PLANNING TEMPLATE**

**This template focuses on the timing of workshop activities and how they related to your learning objectives. It is a good planning and management tool, and an abbreviated version can be used as an agenda to share with participants.**

| **Clock Time** | **Allotted**  **Time** | **Activity** | **Facilitator** | **Comments, learning objectives targeted, slides/handouts** |
| --- | --- | --- | --- | --- |
|  |  |  |  |  |
|  |  |  |  |  |
|  |  |  |  |  |
|  |  |  |  |  |
|  |  |  |  |  |
