# Supplementary material for: Planning and Presenting Workshops That Work: A Faculty Development Workshop
Source: MedEdPORTAL. 2021 May 11;17:11158. doi: 10.15766/mep_2374-8265.11158 (PMC8110637; doi:10.15766/mep_2374-8265.11158)
Supplement: Supplementary file 1 — Facilitator Guide.docxSession Agenda.docWorkshop Slides.pptWorkshop Template Handout.docxAdditional Handout.docxAdvanced Handout.docxSession Evaluation.docx [file mep_2374-8265.11158-s001.zip › E. Additional Handout.docx]

**Designing Workshops That Promote Active Learning and Behavior Change:**

**An Outline for Success**

**Workshop Definition**: “A workshop is a short-term learning experience that encourages active, experiential learning and uses a variety of learning activities to meet the needs of diverse learners.”

(Brooks-Harris JE and Stock-Ward SR from *Workshops: Designing and Facilitating Experiential Learning)*

**When is a workshop not a workshop?** When it is a lecture and the learners are passive participants during the experience.

**Characteristics of an effective workshop**:

1. Active involvement
2. Application of new learning
3. Variety of learning activities
4. Interaction among participants
5. Experiential learning experiences
6. Relevant and practical information
7. Emphasis on problem solving, skill building, or development of competence
8. Presenter as a facilitator rather than a teacher
9. Behavior change as an outcome

**Pre-workshop Planning:** Prior to developing a workshop, you need to collect information and make preliminary decisions. Without having answers to the questions below, you risk the chance that your workshop will not be successful.

1. Collect preliminary data (who, what, when, where and why):
   1. Why is this workshop being requested or offered?
   2. Who will be attending (audience characteristics) and what are the expected numbers?
   3. When will it occur (time of day, length of session)?
   4. What content should be covered and what outcome(s) or main emphasis of the workshop is expected (e.g., problem solving, knowledge acquisition, skill building, development of competence)?
   5. Where will the workshop be conducted (physical space and layout of the room, AV equipment, multimedia and internet capabilities)?
2. Determine if a workshop is the optimal approach. Most teaching is intended to fill an identified gap in learners’ knowledge or performance, so you will need to decide if a workshop is the best way to fill the identified gap. In general, workshops are an excellent way to expose learners to new models or tools, and have a chance to “play with them” and learn their usefulness.
3. Identify what the participants need to know. Assessing or predicting the learning needs of the participants takes time, but avoid over-analyzing the question. Too much analysis is just as bad as too little analysis. Techniques that can be used include the following:
   1. Interviews
   2. Questionnaires
   3. Focus groups
   4. Observation
   5. Analysis of previously collected data
   6. Review of the literature
   7. Brainstorming sessions
   8. Contact with others (programs, individuals, departments etc.)
   9. You can also ask a group a few questions at the beginning of a workshop.
4. Set workshop goals and objectives:
   1. Base your objectives on your analysis of what is needed to fill the gap between current and expected performance.
   2. Defined objectives help you stay on track, keep the session relevant and focused, and let participants know what will be covered during the session.
   3. Articulated objectives serve as a roadmap for the learners to help them determine what they should gain from this training.
5. Identify resources that will enhance the workshop.

**Basic Workshop Framework.** Experiential learning is crucial to an effective workshop. Therefore, we prefer a four-stage model of experiential learning first described by David Kolb:

1. Reflecting on Experience
2. Assimilating and Conceptualizing Information
3. Experimenting and Practicing
4. Planning for Application

Based on this framework, we use the following model to design workshops and maximize learning of all individuals, regardless of their learning style.

1. Introductions/goals and objectives/agenda. (Feedback on the proposed agenda from the participants is helpful in ensuring concordance between your plan and the group's needs)
2. Reflective exercise and/or “ice breaker” – a warm-up exercise that promotes interaction, comfort, and/or reflection
3. Brief didactics - tell them something that they will later practice/apply (5-15 minutes)
4. Active learning engagement such as:
   1. individually think or write
   2. think-pair-share
   3. small-group breakouts
   4. large-group brainstorming
   5. role plays
   6. games
   7. video analysis
   8. case discussions
5. Application - a commitment from participants on how they will incorporate the material learned into their day-to-day practice
6. Wrap-up and evaluation (evaluation allows one to continuously improve the presentation, after identifying what worked and what did not work)

**Additional considerations:**

1. Breaks
2. Sign in sheets
3. CME
4. Food
5. Room configuration
6. Handouts
7. Leave with or send a product
8. Flexibility
9. How to handle questions

**Tips for Developing Workshops:**

Pre-planning

- Start planning early. Developing a workshop takes more time than one might think. Starting early will give you time to pilot the workshop for colleagues and get feedback before you actually offer the workshop for your learners.
- Limit the amount of material covered: less content learned well is better than a lot of content that is not remembered. Provide relevant and practical information.
- Remember principles of adult learning - respect the participants’ previous knowledge and experiences, their motivations to learn, their potential resistance to change, and their abilities to serve as co-learners
- Participants are unique in their needs and learning styles. Vary learning activities and teaching styles to account for different learning styles.
- Invite colleagues to work with you on the project. It will enhance the breadth of experience of the presenters and is a great way to network or mentor others.

Presentation style

- Be conversational in your delivery and create a relaxed atmosphere for learning.
- Be flexible during the delivery of the workshop and allow for changes that arise during the presentation. As important as it is to plan ahead, it is even more important to be prepared to adjust or even abandon your prepared agenda.

Stages of the workshop

- Introduce presenters and participates.
- Use icebreakers to engage the learner from the beginning.
- Ask questions that stimulate problem-solving and critical thinking.
- Urge participants to ask questions.
- Facilitate learning - do not lecture. Keep didactic time to a minimum, and break your training into bite-size chunks.
- Provide opportunities for active listening.
- Enhance learning through experiential activities and practice.
- Have learners teach each other.
- If you use technology, make sure that it meets the needs of the learners. Don’t use technology just because you can.
- Request feedback from the group as to whether you have accomplished your stated objectives and how they would improve the session in the future.
- Have learners review what they have learned.
- Always evaluate your workshop so the next version will be even better.
- Enjoy yourself and have fun!

**Workshop Template based on Kolb’s Learning Cycle**

**Topic** __________________________________________________________________

**Length** ___________ **Target audience** __________________________________

| Goals |  |
| --- | --- |
| Objectives | 1.  2.  3. |
| **Ice breaker/reflection/**  **attention getter** |  |
| **Share/Didactics** |  |
| **Practice/Activities** |  |
| **Apply what was learned (Get the participants to make a commitment to try this at “home” or to reflect on how they can use this)** |  |
| **Summary/Conclusion** |  |

AV equipment needed:

Handouts:

**WORKSHOP PLANNING TEMPLATE**

**This template focuses on the timing of workshop activities and how they related to your learning objectives. It is a good planning and management tool, and an abbreviated version can be used as an agenda to share with participants.**

| **Clock Time** | **Allotted**  **Time** | **Activity** | **Facilitator** | **Comments, learning objectives targeted, slides/handouts** |
| --- | --- | --- | --- | --- |
|  |  |  |  |  |
|  |  |  |  |  |
|  |  |  |  |  |
|  |  |  |  |  |
|  |  |  |  |  |
|  |  |  |  |  |

**Resources:**

1. Steinert Y, Boillat M, Meterissian S, Liben S, McLeod PJ. Developing successful workshops: a workshop for educators. Medical teacher. 2008 Jan 1;30(3):328-30.
2. Tiberius R, Silver, I. Guidelines for conducting workshops and seminars that actively engage participants. University of Toronto, Department of Psychiatry, 2001. Accessed on November 1, 2020 at <https://docuri.com/download/conducting-workshops_59c1e1e8f581710b286a419c_pdf>
3. Steinert Y. Guidelines for Site-Specific Activities: Faculty development principles and strategies. 2006. Accessed on November 1, 2020 at <https://afmc.ca/timg/pdf/SSG_en.pdf>
4. Steinert Y and Boillat M. A Workbook on Designing Successful Workshops. Accessed on November 1, 2020 at <https://www.slideshare.net/mtranvan/designing-workshopswork-workbook>

**References:**

1. Brooks-Harris and Stock-Ward (1999). *Workshops: Designing and facilitating experiential learning.* Thousand Oaks, California SAGE Publications, Inc.
2. McCain DV and Tobey DD (2004). *Facilitation basics*. Alexandria, VA: ASTD.
3. Steinert Y. Twelve tips for conducting effective workshops. *Med Teach*. 1992;14(2-3):127-131.
4. McCarthy B and O'Neill-Blackwell J (2007). *Hold on, you lost me! Use learning styles to create training that sticks.* Alexandria, VA: ASTD.
5. Skeff, KM Stratos FA, et al. (1997). Faculty development: A resource for clinical teachers. *Journal of General Internal Medicine*. 12(S2):S56-S63.
6. Clark RC. (2010) *Evidence-based training methods: A guide for training professionals*. Alexandria, VA: ASTD.
7. Piskurich GM (2003). *Trainer Basics*. Alexandria, VA: ASTD.
8. Stolovitch HD, and Keeps EJ. *Telling ain't training*. Alexandria, VA: ASTD.
9. Jolles RL. (2005) *How to run seminars and workshops: Presentation skills for consultants, trainers and teachers.* 3rd ed. Hoboken, NJ. John Wiley & Sons, Inc.
10. Bland CJ. (1980). *Faculty development through workshops*. Springfield, IL: Charles C. Thomas.
11. Knowles M. (1990). *The adult learner: A neglected species*. 4th ed. Houston: Gulf Publishing Company.
12. Silberman M. (2004). *The best of active training: 25 one-day workshops*. San Francisco, CA. John Wiley & Sons San Francisco, CA. John Wiley & Sons
13. Silberman M. (2007). *The best of active training II: 25 one-day workshops*.
14. Silberman M. (2006) *Training the active training way: 8 strategies to spark learning and change.* San Francisco, CA. John Wiley & Sons.
15. Silberman M and Auerbach C. (2006) *Active training: A handbook of techniques, designs, case examples, and tips.* 3rd edition. San Francisco, CA. John Wiley & Sons.
16. Mitchell, G. (1998). *The trainer's handbook: The AMA guide to effective training* 3rd edition. New York: AMACOM.
17. Interactive Techniques. http://www.fctl.ucf.edu/TeachingAndLearningResources/CourseDesign/Assessment/content/101_Tips.pdf. Accessed April 30, 2011.
18. Johnson D. *Top ten secrets for a successful workshop*. http://www.doug-johnson.com/dougwri/top-ten-secrets-for-a-successful-workshop.html. Accessed April 30, 2011.
19. Angelo TA and Cross KP. (1993) *Classroom Assessment Techniques*. 2nd edition. Jossey-Bass: San Francisco.
20. Silberman M. (1996) *Active Learning: 101 strategies to teach any subject*. Allyn and Bacon: Boston.
21. Skeff K and Stratos G. *Methods for teaching medicine*. (2010). ACP. Teaching Medicine Series.
22. Turner T, Palazzi D, Ward M. The Clinician-Educator's Handbook. MedEdPORTAL: Available from www.mededportal.org ID 7749 and [www.bcm.edu/pediatrics/clinician_educator_handbook](http://www.bcm.edu/pediatrics/clinician_educator_handbook)
