# Supplementary material for: Planning and Presenting Workshops That Work: A Faculty Development Workshop
Source: MedEdPORTAL. 2021 May 11;17:11158. doi: 10.15766/mep_2374-8265.11158 (PMC8110637; doi:10.15766/mep_2374-8265.11158)
Supplement: Supplementary file 1 — Facilitator Guide.docxSession Agenda.docWorkshop Slides.pptWorkshop Template Handout.docxAdditional Handout.docxAdvanced Handout.docxSession Evaluation.docx [file mep_2374-8265.11158-s001.zip › F. Advanced Handout.docx]

**Workshops 201**

1. Always have a contingency plan.
   1. Consider having a plan A and a plan B for any workshop. For example, if you are teaching on curriculum design and you get to the workshop and learn that everyone is an expert, be ready to implement plan B, such as a train-the-trainer model.
   2. Take with you back-up equipment such as VGA adapters and a back-up computer, a remote slide changer, handouts in case the equipment does not work, and screenshots of anything you might need from the internet.
   3. Make sure facilitators are comfortable leading all the parts of the workshop in case one of your facilitators is unable to participate.
2. Think about how your participants can use what they have learned. How can you make it easy for them?
   1. Participants appreciate receiving ready-made materials that they can use at their own institutions.
   2. Resources often can be more helpful than references.
3. Needs assessments often must be on-the-spot for national meetings. For example, PAS does not provide a list of individuals who sign up for your workshop. Make sure to ask the conference organizers if you are unsure.
   1. Remember that other workshop presenters may want participants to do activities before the meeting. Try to send your materials 2 weeks in advance of the meeting – your email will be the first that the participants see and, thus, they may be more likely to do your activity.
   2. Send friendly email reminders about the activity, as you would to get individuals to respond to a survey – 3 emails usually gets the most participation.
4. Introductions of participants are important – when you have more than 12 people in a room, having everyone introduce themselves can take up valuable time. You do, however, want people to feel valued for the knowledge and skills they bring to the table and to feel comfortable working together.
   1. Have individuals introduce themselves within small groups, especially if they will be working together.
   2. Utilize “show of hands” for attributes/characteristics of workshop participants, it can be as simple as having a show of hands of those from the East Coast, West Coast, Midwest etc.
5. Ice breakers are invaluable but should be relevant to the content of your presentation. They should serve as a “warm up” to what you will be doing but not demand deep reflection.
   1. Consider investing in books about ice breakers – they not only have ready-made exercises but also can provide you with ways to adapt an activity to meet your needs.
   2. A great book is *Jolts! Activities to Wake Up and Engage Your Participants* by Thiagarajan S and Tagliati T.
   3. You can also search the internet for “ice breakers” and find hundreds of them freely available.
6. Consider using a flipped-classroom model to teach the didactic portions of your workshop (or at a minimum – the majority of the content and then review salient points during the workshop). This method requires significant thought about how to ensure that the learners will actually do the activity before attending the workshop. Consider
   1. Raffling prizes for those who completed the activity
   2. Organizing the workshop activities to include at least one person in each group who did the activity
   3. Minimizing the amount of learning required outside the workshop – for example, no more than a 20-minute time commitment for a 1.5- to 3-hour workshop.
7. Workshop participants usually enjoy learning from each other, so provide opportunities for people to share their knowledge with others.
8. Consider adapting pre-packaged workshops for your training needs.
   1. Explore what is available on MedEdPORTAL.
   2. A great book is *101 Ways to Make Training Active* by Mel Silberman.
   3. You can also purchase pre-packaged workshops from both Mel Silberman and the American Society for Training and Development (ASTD).
9. If you have a large group of participants in a workshop, it can still be interactive.
   1. Use the poor man’s audience-response system (colored note cards – priceless!).
   2. Provide “seat work” activities that they can work on and then share with someone sitting nearby (as well as the traditional “Think-Pair-Share”)
   3. Include Today’s Meet – a free online sharing system – think of it as a virtual flipchart (or an online flipchart) that participants can use to type their responses to your large-group brainstorming questions [www.todaysmeet.com](http://www.todaysmeet.com)
10. Breaks can be helpful if workshops last more than 2 hours. Remember that individuals need time to process what they have learned as well as an opportunity to use the bathroom.
11. Don’t depend on national meetings to provide you with evaluation data – Take your own evaluations forms specifically to provide you with feedback for crafting the next workshop.
12. Remember and reflect on the Workshop Credo from Mel Silberman:

What I hear, I forget.

What I hear and see, I remember a little.
What I hear, see, ask questions about, and discuss, I begin to understand.
What I hear, see, ask questions about, discuss, and do, I acquire knowledge and skills about.
What I teach to another, I master.
