# Supplementary material for: Planning and Presenting Workshops That Work: A Faculty Development Workshop
Source: MedEdPORTAL. 2021 May 11;17:11158. doi: 10.15766/mep_2374-8265.11158 (PMC8110637; doi:10.15766/mep_2374-8265.11158)
Supplement: Supplementary file 1 — Facilitator Guide.docxSession Agenda.docWorkshop Slides.pptWorkshop Template Handout.docxAdditional Handout.docxAdvanced Handout.docxSession Evaluation.docx [file mep_2374-8265.11158-s001.zip › G. Session Evaluation.docx]

**Planning and Presenting Workshops that Work**

**Evaluation**

1. Was this session informative and useful? ____yes ____no

Comments: ________________________________________________________________________________________________________________________________________________

1. What were the aspects of this activity you found particularly good/helpful?

________________________________________________________________________________________________________________________________________________

1. What are the areas that could be improved?

________________________________________________________________________________________________________________________________________________
