# Supplementary material for: Planning and Presenting Workshops That Work: A Faculty Development Workshop
Source: MedEdPORTAL. 2021 May 11;17:11158. doi: 10.15766/mep_2374-8265.11158 (PMC8110637; doi:10.15766/mep_2374-8265.11158)
Supplement: Supplementary file 1 — Facilitator Guide.docxSession Agenda.docWorkshop Slides.pptWorkshop Template Handout.docxAdditional Handout.docxAdvanced Handout.docxSession Evaluation.docx [file mep_2374-8265.11158-s001.zip › A. Facilitator Guide.docx]

**Planning and Presenting Workshops That Work**

**Facilitator Guide**

Advanced Preparation and Room Set-Up

- The week before the workshop, the facilitators should send an email to the participants to come to the session with an idea for a workshop they would like to develop, in order to maximize the working time.
- The typical room set-up includes round tables for the participants and a front table or podium for the facilitators to use for handouts and the agenda. A flip chart with markers should be placed at the front of the room, and AV equipment for PowerPoint is needed.

Introduction

- Facilitators welcome participants to the workshop. If facilitators and all participants do not know each other already, facilitators should introduce themselves and then ask participants to individually introduce themselves.
- A brief needs assessment is done by show of hands – ask participants to raise their hand if they have ever attended a workshop, and then ask them to raise their hand if they have ever led a workshop.
- Show learning objectives (Appendix C, slide 3) and session agenda (Appendix C, slide 4).

Ice Breaker

- One facilitator asks participants to think about and list characteristics of effective workshops, while the other facilitator notes responses on the flip chart. Make a comment tying responses to the session (e.g. “You have mentioned several characteristics of effective workshops described in the literature. We will be exploring many of these in more detail today.”).

Basic Workshop Didactic

- One facilitator presents slides 7–15 (Appendix C) with the brief overview of a basic workshop framework.
- If many participants are experienced workshop leaders, adjust the presentation by seeking their input on ways to conduct a needs assessment and innovative approaches to didactics, and ask them to share their experiences with promoting active learning during the workshops they have conducted.
- The other facilitator follows the presentation with a “reflection-in-action,” pointing out workshop facilitation techniques that had been used so far (e.g. with a large group, introductions and a quick needs assessment can be accomplished with a show of hands rather than taking the time to have everyone speak; a short, easy ice breaker can be used to help people focus on the topic).

Break Out Session

- Instruct participants that they will now begin the “work” of the “workshop.” They have the option of working alone, in pairs, or in small groups to plan a workshop. Note that working with others may promote feedback and networking, while working alone will allow an individual to further develop an idea they have already thought about. Participants may use the idea they thought of in advance, work with others on a different idea, or use the topic of feedback during this exercise.
- Ask for volunteers to announce their workshop idea so that participants can arrange themselves in working groups based on interest. A facilitator may record these topics on the flip chart.
- Display slide 16 (Appendix C) during the break out session for reference.
- Hand out the blank workshop template (Appendix D) for participants to use during their work.
- Offer the detailed agenda for this session as an example if desired (Appendix B).
- Facilitators walk around the room to answer questions and help guide the participants in their work.
- After the individual and small group work, facilitators conduct a large group debriefing:
  - Ask for participants to share insights and challenges from this activity.
  - Encourage participants to continue to work on their ideas after this session and to consider submitting a workshop proposal to a national meeting.
  - Facilitators may offer to review proposals and provide feedback if participants desire.

Wrap-Up

- One facilitator presents Top 10 Tips for Conducting Workshops (Appendix C, slides 17-21).
- The other facilitator follows with a “reflection-in-action,” noting the importance of hands-on activities during a workshop and the techniques utilized for the large group debriefing.
- Ask participants for any final questions about planning and conducting workshops.
- Commitment to action – ask participants to write down one new thing they plan to do as a result of this session, noting that people are more likely to achieve a goal if they write it down.
- Provide additional handouts (Appendices E and F) to participants to take home.
- Ask participants to fill out the session evaluation (Appendix G).
