# Supplementary material for: Planning and Presenting Workshops That Work: A Faculty Development Workshop
Source: MedEdPORTAL. 2021 May 11;17:11158. doi: 10.15766/mep_2374-8265.11158 (PMC8110637; doi:10.15766/mep_2374-8265.11158)
Supplement: Supplementary file 1 — Facilitator Guide.docxSession Agenda.docWorkshop Slides.pptWorkshop Template Handout.docxAdditional Handout.docxAdvanced Handout.docxSession Evaluation.docx [file mep_2374-8265.11158-s001.zip › Educational_Summary_Report_11158.pdf]

MedEdPORTAL® | The AAMC Journal of Teaching and Learning Resources

Original Publication Open Access

Planning and Presenting Workshops That Work: A Faculty Development Workshop

Elisa A. Zenni, MD*, Teri L. Turner, MD, MPH, MEd

*Corresponding author: elisa.zenni@jax.ufl.edu

Abstract

Introduction: Workshops are commonly used in higher education, although faculty often have little or no training in how to develop and deliver this type of teaching methodology. This publication can be used to deliver a 1-hour active learning session to faculty utilizing experiential learning as a framework. **Methods:** An hour-long workshop on developing and implementing effective workshops was given to five cohorts of participants in the Academic Pediatric Association's Educational Scholars Program (ESP) between 2010 and 2018, following a 2008 pilot. After a brief didactic presentation, participants developed their own workshop plans. A unique reflection in action was utilized to model effective workshop facilitation techniques. Written surveys were used to evaluate the effectiveness of the workshop. Data from the ESP graduates were used to report the percentage of respondents who conducted their own workshop postgraduation. **Results:** A total of 116 faculty participated in this workshop over the course of 5 years. One hundred and fourteen participants stated they found the session to be useful. The role modeling/reflection by the facilitators and the opportunity to work with others on a workshop plan were described as the most valuable aspects. Approximately 70% of scholars who responded to postgraduation surveys had conducted at least one regional, national, or international workshop. **Discussion:** This faculty development session implements active and adult learning principles to model and teach participants how to develop and lead an effective workshop. It also provides a mechanism for collaboration among participants to develop a workshop based on shared interests.

Keywords
Workshop, Education, Active Learning

Educational Objectives

By the end of this activity, learners will be able to:

1. Describe three to five characteristics of effective workshops that promote active learning and behavior change.
2. Develop a workshop outline using experiential learning theory as a framework for design and delivery.

Introduction

Workshops are commonly used in higher education as a method to promote change in knowledge, attitudes, and skills, particularly in faculty development.[1-4] Despite this popularity, many faculty have had little or no training in how to leverage active learning strategies to engage participants and promote behavior change. The vast majority of the work on developing successful workshops has been performed by Steinert and colleagues.[5-7] In 2008, Steinert and her team published a short communication on the results of a workshop for educators on this topic.[4] Beyond self-perceived efficacy data, 64% of participants had conducted a workshop in their own setting by 9 months after the session. Our *MedEdPORTAL* publication builds off the work by Steinert et al. and utilizes theories and conceptual frameworks from higher education to provide guidance on this topic. Steinert has also deduced five important characteristics of effective faculty development workshops: the use of experiential learning, the provision of feedback, effective peer and colleague relationships, the application of principles of teaching and learning, and the use of multiple instructional methods.[3,8]

Citation:
Zenni EA, Turner TL. Planning and presenting workshops that work: a faculty development workshop. *MedEdPORTAL*. 2021;17:11158.
https://doi.org/10.15766/mep_2374-8265.11158

A search of *MedEdPORTAL* publications revealed submissions on teaching to promote active learning[9] and to train teachers to be small-group facilitators.[10] To date, there have been no publications in *MedEdPORTAL* focused on teaching faculty the skills for designing and delivering a workshop.

Copyright © 2021 Zenni and Turner This is an open-access publication distributed under the terms of the Creative Commons Attribution license.

Designing effective workshops consists of three basic areas: preworkshop planning, the workshop itself, and evaluating whether or not learning has occurred. This workshop applies the principles of both experiential and adult learning. The framework of the workshop models the four stages of Kolb's experiential learning cycle[11,12]:

1. Reflecting on experience (reflecting on what the learner already knows),
2. Assimilating and conceptualizing information (enriching or expanding on existing knowledge),
3. Experimenting and practicing (trying out new knowledge and skills), and
4. Planning for application (obtaining a commitment from the learner to change practice).

Regardless of the quality of the presentation, research has suggested that adults learn primarily when they have a need to learn.[13] Adults tend to be motivated to learn when their own experiences are acknowledged and leveraged, the content is relevant to the work they do, learning is authentic, and the learners have some control over the learning experience.[14] Data also suggest that active learning is better than passive learning.[15] Effective workshops utilize this evidence to promote active learning that results in acquisition of skills.

The purpose of this educational activity is to enable participants to describe characteristics of effective workshops and to develop a workshop outline using experiential learning theory as a framework for design and delivery. The target audience for this session is primarily junior and mid-career faculty who have never conducted a workshop. However, anyone from medical school to fellowship can learn or hone the skills necessary to develop and deliver a successful workshop. This learning activity is meant to close the skill gap for those unfamiliar with the steps in the process. To gain an understanding of our participants' knowledge and skills on designing and delivering workshops, we gathered data from a targeted sample of participants prior to the development of the first workshop session, asked questions related to experience in delivering a workshop during each session, and analyzed feedback from previous iterations of the session to inform the current version of the workshop. We also conducted an informal needs assessment with 10 faculty members from both large and small institutions whose educational focus at their institutions was faculty development. From these conversations evolved the top tips for developing a workshop.

Methods

Curricular Context

We created a 1-hour workshop to teach participants how to develop and implement effective scholarly workshops. After a pilot session in 2008 with more than 30 participants, we presented the session five times between 2010 and 2018 as part of the curriculum for the Academic Pediatric Association's Educational Scholars Program (ESP), a 3-year national faculty development program for academic pediatric educators wishing to build their skills in educational scholarship and leadership. Part of the ESP curriculum included an in-person, daylong ESP Day session at the beginning of the annual Pediatric Academic Societies' meeting. We delivered this session on planning and presenting workshops as part of the scholarly dissemination portion of the longitudinal curriculum.

Since the session was part of a larger curriculum, we were charged to deliver it in a 1-hour time frame. In building upon the work by Steinert, we found that the most successful component was the active learning. Thus, we minimized the didactic delivery of content and added the reflection in action to allow participants to understand why we had chosen to do or not do various activities and how these impacted participant learning. We gave each presentation to a different group of participants. Based on feedback from the first 4 years of implementation, we increased the duration of the session to 1 hour and 15 minutes for the last 2 years. Thirty-three participants attended in 2010, 15 in 2014, 24 in 2015, 22 in 2017, and 22 in 2018.

Implementation

Optimal physical setup for this workshop included roundtables for the participants, along with a flip chart and equipment for the PowerPoint. Two facilitators who had experience in leading workshops locally and nationally led each session. Facilitators presented from the front of the room and circulated during the active learning portion of the session to be able to provide more individualized guidance independent of the size of the overall group. We asked participants in advance to come to the session with an idea for a workshop they would like to develop in order to maximize the use of their working time.

The facilitator guide (Appendix A) describes the activities of the workshop. Table 1 details the recommended time line for the session. After introduction of the facilitators, we conducted a brief needs assessment of the participants by show of hands. We asked participants to raise their hands if they had ever attended a workshop and then asked them to raise their hands if they had led a workshop. Personal introductions were not necessary in this

Copyright © 2021 Zenni and Turner This is an open-access publication distributed under the terms of the Creative Commons Attribution license.

Table 1. Recommended Time Line for 75-Minute Session

Activity	Allotted Time	Clock Time
Introduction (facilitator introduction, needs assessment, goals and objectives, agenda)	5 minutes	0:00
Icebreaker (characteristics of effective workshops)	5 minutes	0:05
Basic workshop didactic (including 1 minute of reflection)	10 minutes	0:10
Breakout sessions (planning a workshop)		
Planning a workshop—group work	25 minutes	0:20
Large-group debrief (group workshop plans)	15 minutes	0:45
Wrap-up		
Top 10 tips didactic	5 minutes	1:00
Reflection	1 minute	1:05
Questions	5 minutes	1:06
Commitment to action	4 minutes	1:11

context, as the participants already knew each other. Beginning in 2017, we included a Plan B in our agenda (Appendix B), with minor modifications for groups with predominantly experienced workshop leaders.

After review of the goals, objectives, and session agenda (Appendix C, slides 3-4), we conducted an icebreaker to engage participants in thinking about workshops (Appendix C, slides 5-6). One facilitator asked the group to list characteristics of effective workshops, while the other facilitator noted the responses on the flip chart. We tied the group's responses to things we would be discussing during the session (e.g., "You have mentioned several characteristics of effective workshops described in the literature. We will be exploring many of these in more detail today").

Next, one facilitator presented a brief didactic with a PowerPoint presentation on a basic workshop framework (Appendix C, slides 7-15), followed by a reflection in action from the other facilitator. The purpose of the reflection was to overtly point out techniques for facilitating workshops already used during the current session, so that participants could clearly see the content in action. For instance, we noted that with a large group, introductions could be accomplished by show of hands rather than taking the time to have everyone speak. We also mentioned the use of a short icebreaker to get people thinking about the topic.

Then, we instructed participants to actively plan a workshop and gave them the flexibility to work alone, in pairs, or in small groups in breakout sessions. We offered participants the option of using the idea they had thought of in advance, working with one or more colleagues on another idea, or using the topic of feedback to gain the hands-on experience of planning a workshop. We found it helpful to ask for volunteers to announce their topic ideas so that participants could arrange themselves in working groups based on interest. We displayed a PowerPoint slide with tips to guide the participants (Appendix C, slide 16) as they planned their proposed workshops. In addition, we provided a workshop template handout (Appendix D) as a working tool and offered our detailed agenda for this session (Appendix B) as an example. We walked around the room during this time to answer questions and help guide the participants in their work.

We then conducted a large-group debriefing to discuss insights and challenges from the activity. We encouraged the participants to continue to work on their ideas after the session and to consider submitting a workshop proposal to a national meeting.

The final portion of the session was the wrap-up, which consisted of a presentation of the top 10 tips for conducting workshops (Appendix C, slides 17-21), a reflection in action by a facilitator (e.g., the importance of hands-on activities in a workshop, techniques utilized for large-group debriefings), final questions from the participants, and a commitment to action in which the participants were asked to write down one new thing they planned to do as a result of this session. We provided participants with an additional handout to use after the session (Appendix E). We added another handout (Appendix F) in 2018, as we observed that some participants came with experience in implementing workshops over time and sought more advanced information.

The session evaluation (Appendix G), developed by the ESP evaluation team, was part of the standard written ESP Day evaluation. We evaluated learners' reactions to the sessions by written, anonymous, end-of-day evaluations, with a 100% response rate each year. The evaluation asked participants if the session was informative and useful (yes/no answer with option of writing comments), the aspects they found to be particularly good/helpful (narrative answer), and the areas they thought could be improved (narrative answer). The ESP evaluation team provided us with the aggregated evaluation data for this session, and we reviewed the narrative responses for trends. A previous publication of the outcomes of ESP graduates from 2009 to 2013[16] and unpublished data from a survey of ESP graduates from 2015 to 2019 were reviewed for the percentage of graduates who had delivered workshops.

This workshop was evaluated using two of the four levels of the Kirkpatrick model of program evaluation,[17] participant satisfaction (Level 1) and participant behavior (Level 3).

Results

Participant Satisfaction

One hundred and fourteen of the 116 total participants stated that they found the session to be useful. The two respondents

Copyright © 2021 Zenni and Turner This is an open-access publication distributed under the terms of the Creative Commons Attribution license.

who stated that it was not useful, one from 2014 and one from 2015, noted that they already had experience in leading workshops. Respondents noted numerous aspects of the session as being particularly good, most commonly the role modeling/reflection by the facilitators and the opportunity to work with others to begin to develop a workshop plan. The most common suggestion for improvement was lengthening the session to give more time to the breakout portions. The Figure

**Enablers of learning**<br>**"What worked well?"**	**Inhibitors of learning**<br>**"What could be improved?"**
Modeling and reflection on action	More structure/explicit instructions
• "Really astutely done in a metacognitive way. Doing a workshop on a workshop, and reflecting on it while doing it. Very helpful."<br>• "Modeling an effective workshop was super helpful."<br>• "I really liked the part of how the facilitators 'thought out loud' on the steps they employed with how to organize a successful workshop. Very effective approach."	• "Maybe more structure during the meeting and planning a workshop to keep us on task with the actual workshop planning as we spent the whole time brainstorming because we did not realize we were supposed to use the worksheet."<br>• "The part where we went around the room and found someone with similar workshop interest caught me off guard. Perhaps if I had been primed, it would have been helpful."
Active learning techniques	More ideas for active learning techniques
• "Love that we were told to come with workshop ideas, but also that we were given time (and encouraged) to come up with ideas as a group, and given the freedom to work with whoever we wanted on whatever topic we wanted. I think this really increases the chances of some of these workshop ideas coming to fruition. Very practical and helpful."<br>• "Amazing to have a brainstorming session and come up with the skeleton for a workshop in less than an hour."<br>• "The short didactic, with lots of group activity was very effective at keeping me engaged. Time to brainstorm our own workshop was great."<br>• "Loved the active learning."	• "More specific ideas for audience engagement with examples."<br>• "Perhaps consider a very brief demo of 'engaging' techniques (i.e., show audience response system modalities, how to incorporate a video clip into a PPT, example of a fun ice breaker, etc.)—if time allows."<br>• "More ideas on how to make things interactive."
Collaborative group work	More time
• "'Forcing' us to come up with an idea together and make a plan for a workshop was great."<br>• "Opening the door to collaboration was wonderful."<br>• "Allowing the time to actually collaborate and come up with a workshop idea with peers."<br>• "Connecting with co-participants on developing a workshop."	• "Longer time for collaborative workshop development."<br>• "Would have loved another 30 min in this workshop! (or even an hour)."<br>• "A little more time dedicated to what reviewers are looking for in workshops."<br>• "Spend a little more time on how to deal with challenges in workshops—like for example when someone hijacks the conversation."
Flexibility in content delivery	Content level too basic or too advanced
• "Fabulous facilitators from whom there is so much to learn (including watching them immediately switch to plan B in their presentation after learning that most of us had already participated in and led workshops ourselves)—this was key as it became a different workshop that we could still learn from vs. one that had basics that most of us already knew."<br>• "The facilitators were flexible and on-point."<br>• "Love that we were told to come with workshop ideas, but also that we were given time (and encouraged) to come up with ideas as a group, and given the freedom to work with whoever we wanted on whatever topic we wanted. I think this really increases the chances of some of these workshop ideas coming to fruition."	• "A little generic."<br>• "Maybe divide up the room into two groups with one group for those who have never put together a workshop and another for more seasoned ones."<br>• "I have no experience in creating a workshop. I understand a great majority of the scholars have led a workshop previously and so the content of the lecture was changed to adapt to this. However, I felt a bit lost and overwhelmed since I had only superficial knowledge of some of the information being presented in the lecture."

Figure. Example participant comments about enablers and inhibitors of learning.

Copyright © 2021 Zenni and Turner This is an open-access publication distributed under the terms of the Creative Commons Attribution license.

lists narrative comments from the participants, grouped into similar response categories, related to delivery of the workshop content.

Participant Behavior
Data from the previous publication of the outcomes of ESP graduates from 2009 to 2013 (response rate: 76%) indicated that a mean of 5.7 national peer-reviewed workshops (range: 0-15) had been given and that 74% of respondent graduates had delivered at least one national peer-reviewed workshop.[16] Participants who graduated from ESP in 2015-2019 were surveyed for a separate study in 2019 (unpublished data). Of those graduates, at least 1 year postgraduation (graduation years 2017-2018), 45% responded (17 out of 39). Sixty-five percent had conducted at least one regional, national, or international workshop with another ESP colleague ($M = 2.24$, range: 0-15).

Discussion

We presented a faculty development workshop on how to develop and lead workshops to 116 participants over the course of 5 years, utilizing active and adult learning principles to model and teach. Based on Kolb's experiential learning cycle framework, participants were given the opportunity to actively apply the content. Similar to a play within a play, the reflections in action provided a unique opportunity to demonstrate and highlight workshop facilitation techniques. Evaluations revealed high learner satisfaction and later success with implementation of workshops. Lessons learned from developing, implementing, and evaluating this workshop are detailed in Table 2.

Table 2. Tips and Lessons Learned for Facilitators

Tip	Lessons Learned
Be flexible.	Participants bring varying levels of experience to the session, and facilitators should be prepared to adjust the content based on the results of the needs assessment. If the majority of attendees are inexperienced workshop facilitators, the participants with more expertise can be engaged by sharing their experiences and challenges. This flexibility was important during the session, as well as in making modifications to the workshop over time.
Ask participants to come prepared with a topic in mind.	There are benefits and challenges to assigning participants a workshop topic to develop versus allowing them to work on a topic of their choice. Although some participants may not have an idea and, therefore, use most of their working time to come up with a topic, those who do work on developing their own idea appear to be more successful in ultimately turning that idea into a workshop. Facilitators can encourage participants in advance to arrive with a topic in mind but should be prepared with backup topics for those who need suggestions.
Facilitate future collaborations.	The opportunity to work with colleagues with shared interests led to future collaboration on development of workshops. Groups that collaborated following initial workshops were used as examples of success stories for subsequent workshops.
The more work time, the better.	We received consistent feedback that 1 hour, and even 1 hour and 15 minutes, was not enough time for this session. Participants want more time to work together and to receive feedback on their work.

The generalizability of this resource may be limited by our experience with a special audience. The participants in the ESP have dedicated their careers to educational scholarship and may have had prior knowledge or skills in adult learning theory and effective teaching techniques. A different audience may need more instruction on educational principles. The limitations of our evaluation approach are fourfold. First, the evaluation of this session was a part of a larger evaluation process for the entire curriculum. We were limited in obtaining a more in-depth analysis of individual components of the workshop, such as specific techniques that worked to enable participant learning. This information was gleaned from participants' comments. Second, we relied on participant recall of the past to obtain data on outcomes, and this too was part of a larger evaluation of the program as a whole. Third, we did not conduct qualitative interviews of the participants and could not delve deeper into participant meaning of comments. We also did not do a thematic analysis of the comments but did group them into categories based on our assessment of the participant narratives. Fourth, we did not have data on participants' knowledge and skills in workshop development or implementation and delivery other than the needs assessment conducted at the beginning of each session, which impacted the interpretation of the outcomes of the evaluation. In addition, the participants' success with later implementation of workshops might not be directly attributed to this session.

Leading workshops, especially if peer-reviewed and taught nationally, is an important source of scholarship for medical educators. It is critical for faculty to develop this skill and to teach others how to do it well, just as is done for conducting research and writing abstracts and manuscripts. Many professional medical conferences offer workshops, but they often do not have clear guidelines for submission or peer review. An expected standard for development of workshops could help elevate the rigor and value of this type of educational scholarship.

Appendices

A. Facilitator Guide.docx

B. Session Agenda.doc

Copyright © 2021 Zenni and Turner This is an open-access publication distributed under the terms of the Creative Commons Attribution license.

C. Workshop Slides.ppt

D. Workshop Template Handout.docx

E. Additional Handout.docx

F. Advanced Handout.docx

G. Session Evaluation.docx

All appendices are peer reviewed as integral parts of the Original Publication.

Elisa A. Zenni, MD: Professor and Associate Dean for Educational Affairs, Department of Pediatrics, University of Florida College of Medicine – Jacksonville; ORCID: https://orcid.org/0000-0002-0203-9457

Teri L. Turner, MD, MPH, MEd: Professor, Assistant Dean for Graduate Medical Education, Vice Chair of Education, and Martin I. Lorin Endowed Chair in Medical Education, Department of Pediatrics, Baylor College of Medicine and Texas Children's Hospital

Disclosures
None to report.

Funding/Support
None to report.

Ethical Approval
Reported as not applicable.

References

1. Wilkerson L, Irby DM. Strategies for improving teaching practices: a comprehensive approach to faculty development. *Acad Med*. 1998;73(4):387-396. https://doi.org/10.1097/00001888-199804000-00011
2. Steinert Y. Faculty development in the new millennium: key challenges and future directions. *Med Teach*. 2000;22(1):44-50. https://doi.org/10.1080/01421590078814
3. Steinert Y, Mann K, Centeno A, et al. A systematic review of faculty development initiatives designed to improve teaching effectiveness in medical education: BEME Guide no. 8. *Med Teach*. 2006;28(6):497-526. https://doi.org/10.1080/01421590600902976
4. Steinert Y, Boillat M, Meterissian S, Liben S, McLeod PJ. Developing successful workshops: a workshop for educators. *Med Teach*. 2008;30(3):328-330. https://doi.org/10.1080/01421590801948059
5. Steinert Y. Twelve tips for conducting effective workshops. *Med Teach*. 1992;14(2-3):127-131. https://doi.org/10.3109/01421599209079478
6. Steinert Y, Nasmith L, Daigle N. A faculty development workshop on "developing successful workshops." *Acad Med*. 2000;75(5):554-555. https://doi.org/10.1097/00001888-200005000-00086
7. Steinert Y. Faculty development: from workshops to communities of practice. *Med Teach*. 2010;32(5):425-428. https://doi.org/10.3109/01421591003677897
8. de Grave W, Zanting A, Mansvelder-Longayroux DD, Molenaar WM. Workshops and seminars: enhancing effectiveness. In: Steinart Y, ed. *Faculty Development in the Health Professions: A Focus on Research and Practice*. Springer; 2014:181-195.
9. Li L, Brieger K, Markovitz A, et al. Facilitating facilitation: how to promote active learning. *MedEdPORTAL*. 2014;10:9782. https://doi.org/10.15766/mep_2374-8265.9782
10. Fulton T, Burke C, Hyland K, Kruidering-Hall M, Masters S. Workshop in a box: visual demonstration of small-group facilitation techniques for faculty development. *MedEdPORTAL*. 2010;6:5103. https://doi.org/10.15766/mep_2374-8265.5103
11. Kolb DA. *Experiential Learning: Experience as the Source of Learning and Development*. 2nd ed. Pearson; 2015.
12. Armstrong E, Parsa-Parsi R. How can physicians' learning styles drive educational planning? *Acad Med*. 2005;80(7):680-684. https://doi.org/10.1097/00001888-200507000-00013
13. Piskurich GM. *Trainer Basics*. American Society for Training & Development Press; 2003.
14. Kaufman DM. Applying educational theory in practice. *Br Med J*. 2003;326(7382):213. https://doi.org/10.1136/bmj.326.7382.213
15. Prince M. Does active learning work? A review of the research. *J Eng Educ*. 2004;93(3):223-231. https://doi.org/10.1002/j.2168-9830.2004.tb00809.x
16. Jerardi KE, Mogilner L, Turner T, Chandran L, Baldwin CD, Klein M. Investment in faculty as educational scholars: outcomes from the national Educational Scholars Program. *J Pediatr*. 2016;171:4-5.E1. https://doi.org/10.1016/j.jpeds.2015.12.052
17. Kirkpatrick DL, Kirkpatrick JD. *Evaluating Training Programs: The Four Levels*. 3rd ed. Berrett-Koehler; 2006.

Received: December 1, 2020
Accepted: March 18, 2021
Published: May 11, 2021

Copyright © 2021 Zenni and Turner This is an open-access publication distributed under the terms of the Creative Commons Attribution license.
