# Supplementary material for: Planning and Presenting Workshops That Work: A Faculty Development Workshop
Source: MedEdPORTAL. 2021 May 11;17:11158. doi: 10.15766/mep_2374-8265.11158 (PMC8110637; doi:10.15766/mep_2374-8265.11158)
Supplement: Supplementary file 1 — Facilitator Guide.docxSession Agenda.docWorkshop Slides.pptWorkshop Template Handout.docxAdditional Handout.docxAdvanced Handout.docxSession Evaluation.docx [file mep_2374-8265.11158-s001.zip › B. Session Agenda.doc]

**Planning and Presenting Workshops That Work**

(“Plan B” notes are in red)

| Time | Activities | Facilitator(s) | Comments |
| --- | --- | --- | --- |
| 5 minutes | Introduction   - Needs assessment by show of hands - Goals & objectives - Agenda |  | How many have attended a workshop?  How many have led a workshop?  Mental note that if many participants are experienced workshop facilitators, may need to move people a little to ensure at least 1 experienced facilitator at each table for breakout sessions. |
| 5 minutes | Ice Breaker – characteristics of effective workshops |  | Suggest that participants think about workshops they have attended |
| 10 minutes | Basic workshop framework – brief didactic followed by reflection in action |  | Plan A for predominantly inexperienced workshop leaders – review basics on slides  Plan B for experienced workshop leaders – include challenges for needs assessment and innovative didactics (flipped classroom); participants share their experiences for active learning work and additional considerations |
| 40 minutes | Break-out sessions   - 25 minutes for small group work - 15 minutes for reports |  | Each group (or subgroup) plans a 3-hour workshop on a topic using slide with questions and template handout as guides (offer handout of our detailed work plan as an example if needed; participants were asked in advance to come with ideas)  Report out – focus on each component of workshop, what activity various groups chose and why, insights, challenges |
| 15 minutes | Wrap-up  5 minutes for Top 10 Tips  5 minutes for reflection and commitment   - Reflection - Commitment – write down 1 new thing they plan to do and some share if time   5 minutes for questions  Evaluation |  | Mention that some previous participants have submitted and presented subsequent national workshops  Mention more detailed handout will be available after the session |
